# Case Report: Surgical correction of pulmonary artery sling causing tracheal obstruction in infancy: rapid and complete recovery

**DOI:** 10.3389/fcvm.2025.1558779

**Published:** 2025-03-06

**Authors:** Hüseyin Sicim, Daniel A. Velez, Mohamad Alaeddine

**Affiliations:** Department of Congenital Cardiac Surgery, Phoenix Children’s Hospital, Phoenix, AZ, United States

**Keywords:** pulmonary sling, surgical correction, tracheal obstruction, respiratory failure, infance

## Abstract

Pulmonary artery sling (PAS) is an uncommon congenital anomaly in which the left pulmonary artery (LPA) originates abnormally from the posterior aspect of the right pulmonary artery (RPA). The LPA then traverses between the trachea and esophagus, resulting in compression of the lower trachea. This compression can lead to respiratory symptoms, including wheezing and stridor, and in some cases, airway obstruction. In addition, bronchial compression—commonly affecting the right bronchus—can result in air trapping, pneumonia, and atelectasis. In this report, we present postoperative rapid relief of a symptomatic case in which LPA transection and reimplantation to the main pulmonary artery were performed successfully on cardiopulmonary bypass.

## Case presentation

**Figure 1:**
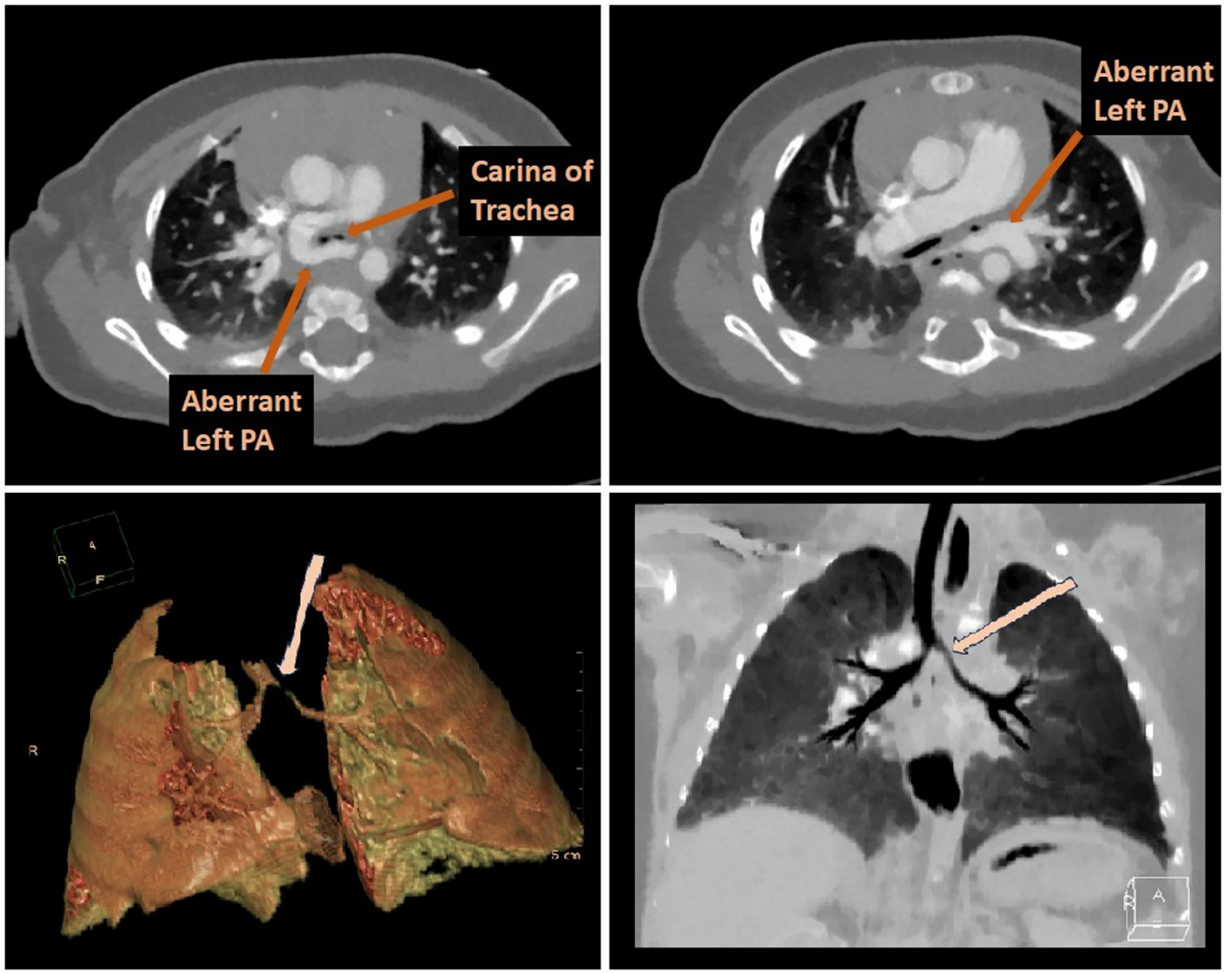
The CT scan confirmed that the left pulmonary artery was originating from the right pulmonary artery and coursing behind the trachea, leading to tracheal compression.

**Figure 2:**
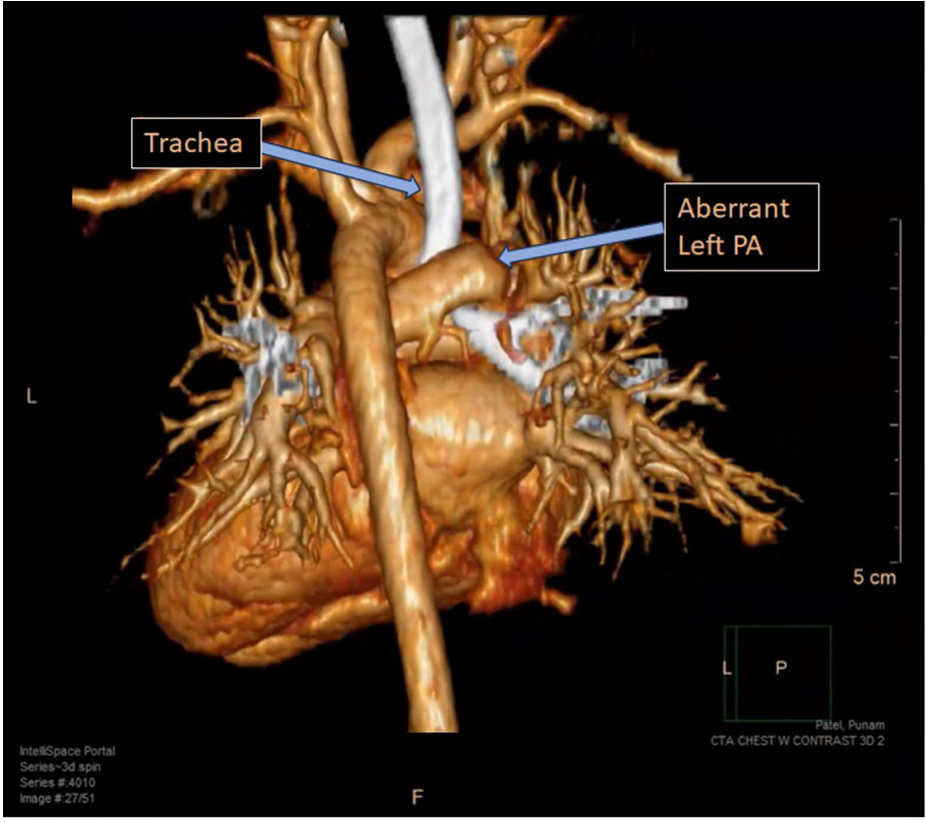
Pulmonary artery sling is illustrated in 3D computed tomography.

**Figure 3:**
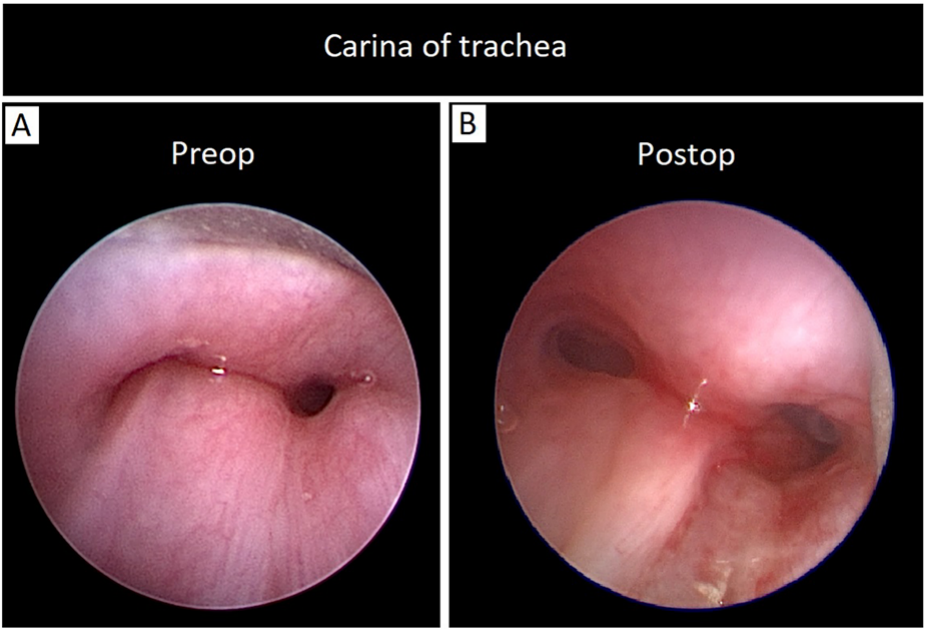
**(A)** Bronchoscopy prior to surgery revealed significant external compression of the trachea at the carina, **(B)** postoperative bronchoscopy in the operating room, showed a marked and satisfactory improvement in the tracheal compression.

A six-month-old female infant was referred to our center with a history of dyspnea and recurrent upper respiratory infections over the last several months. The patient also exhibited stridor and worsening respiratory effort. There was no medical disease, familial history, psychosocial, or genetic pathology in the patient's background and she had no previous diagnosis. A pulmonary sling was detected on transthoracic echocardiography (TTE) in our own center. CT scan was preferred over an MRI because of its superior visualization of vessels and faster scan time. It confirmed that the left pulmonary artery was originating from the right pulmonary artery and coursing behind the trachea, leading to tracheal compression, although no abnormalities were detected within the trachea itself (Figure 1). Echocardiography showed no signs of congenital cardiac defects. Preoperative assessment findings are illustrated in 3D computed tomography (Figure 2). Bronchoscopy prior to surgery revealed significant external compression of the trachea at the carina (Figure 3A). The case was reviewed by a multidisciplinary team, and a decision to proceed with surgery was made.

**Figure 4:**
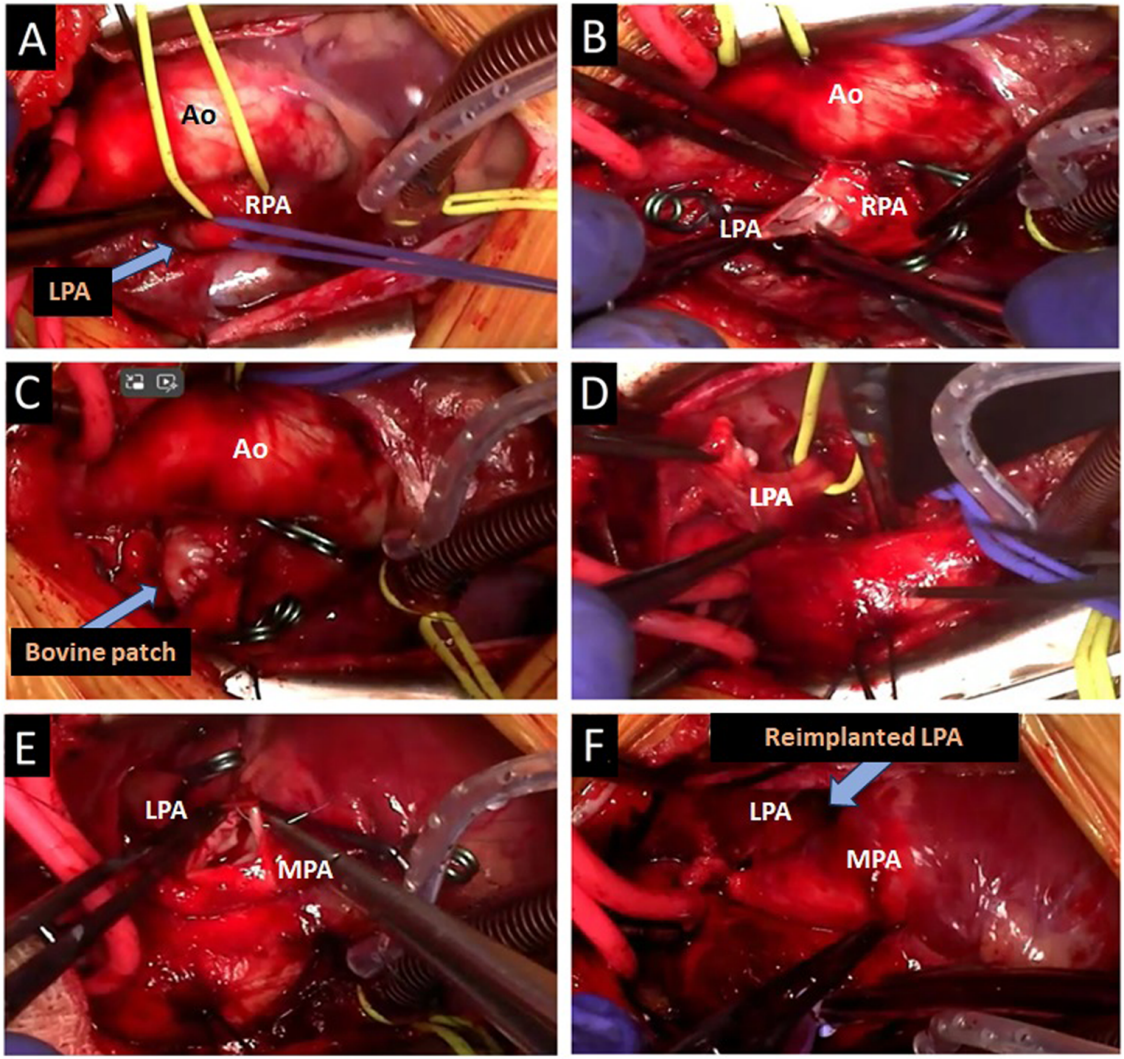
Surgical steps of the aberrant left pulmonary artery reimplantation to the MPA. **(A)** LPA originating from RPA, **(B)** separation of LPA from RPA, **(C)** patchplasty of RPA with bovine pericardium, **(D)** relocation of LPA behind the aorta to the left, **(E)** LPA to MPA anastomosis, **(F)** re-implanted LPA on MPA.

A median sternotomy was performed to address the condition. Intraoperative findings revealed that the LPA arose abnormally from the RPA and passed posteriorly to the trachea. Additionally, a patent ductus arteriosus (PDA) was identified. The PDA was ligated and divided in two stages. Aorto-right atrial cannulation was established, and the left pulmonary artery was reimplanted under cardiopulmonary bypass. The left PA was clamped and detached from its abnormal origin, and the RPA was repaired using a bovine pericardial patch. The LPA was repositioned anterior to the trachea and successfully reimplanted into the main pulmonary artery (Figure 4). After LPA re-implantation, flow and gradient were evaluated only with intraoperative transesophageal echocardiography (TEE) and it was confirmed that the LPA anastomosis was widely open and there was no gradient in the pulmonary arteries. The surgery proceeded without complications. Postoperative bronchoscopy, conducted in the operating room, showed a marked and satisfactory improvement in the tracheal compression (Figure 3B). Following the procedure, she was successfully extubated in OR, and then the patient was transferred to the cardiovascular intensive care unit (CVICU).

The patient remained hemodynamically stable in CVICU. Her condition improved steadily, and she was discharged on the sixth postoperative day. The patient was evaluated in the outpatient clinic one week after discharge. Follow-up clinical assessment revealed no significant symptoms. Transthoracic echocardiography revealed that the LPA anastomosis was widely open and there was no gradient in the pulmonary arteries.

## Discussion

The pulmonary artery sling is a rare congenital malformation in which the left pulmonary artery originates abnormally from the posterior aspect of the right pulmonary artery. From this position, the anomalous left pulmonary artery ascends towards the left lung hilum, passing through the right mainstem bronchus before curving leftward, posterior to the trachea or carina and anterior to the esophagus. This anatomical distortion can lead to upper airway symptoms by compressing both the right mainstem bronchus and the lower trachea (1). If left undiagnosed or untreated, these conditions may contribute to severe complications, including sudden death in neonates and infants (2). The first successful surgical repair of PA sling was performed by Willis Potts and colleagues in 1953, who divided the LPA near its origin, repositioned it anteriorly to the trachea, and re-anastomosed it to the main pulmonary artery (3). Although surgical techniques have remained largely unchanged, the approach has evolved to also address any concurrent tracheal or cardiac anomalies.

Echocardiography is considered the primary diagnostic test for pulmonary artery sling. This non-invasive imaging method is effective in identifying associated intracardiac abnormalities and is safe for critically ill neonates who may have compromised airways. For definitive diagnosis, magnetic resonance imaging (MRI) or computed tomography (CT) with contrast is often used as the gold standard. A characteristic anterior pulsatile indentation of the esophagus seen on a barium swallow study is typically diagnostic for PAS, although this test is not suitable for critically ill neonates, particularly those dependent on mechanical ventilation. Once the diagnosis is confirmed, surgical intervention is recommended for affected patients.

Medical management is primarily supportive until surgical correction is feasible for the patients with PAS. Those on ventilators who experience airway obstruction or pneumonia may require hospitalization. In infants where there is no airway obstruction and symptoms are mild, surgery may not be immediately necessary. The most widely accepted surgical procedure is re-implantation of LPA onto the MPA via sternotomy. Although Susanti et al. (4), reported a case of pulmonary artery sling repair performed without cardiopulmonary bypass, the generally accepted method for PAS repair involves sternotomy with the use of cardiopulmonary bypass. This approach facilitates a more controlled dissection of the left pulmonary artery and allows safe reimplantation into the main pulmonary artery after complete removal of any residual ductal tissue. Performing the surgery with the lungs deflated improves access to the displaced left pulmonary artery, reduces the risk of anastomotic tension, and may improve long-term patency of the left pulmonary artery. Additionally, cardiopulmonary bypass allows for the simultaneous repair of any coexisting congenital heart defects, if necessary.

Although the most used method is reimplanting the left pulmonary artery without tracheal manipulation; the optimal approach often depends on the severity of the tracheal stenosis present alongside the sling. In cases of severe tracheal ring or tracheal stenosis where recovery cannot be achieved, the addition of tracheal surgical methods as a more aggressive approach to LPA reimplantation may be inevitable. These alternative surgical techniques for PSA include tracheal translocation, tracheal resection, slide tracheoplasty and patch tracheoplasty. One of these alternative surgical method for PAS repair was proposed by Jonas et al. (5). This technique involves translocating the undivided left pulmonary artery to a position anterior to the trachea during tracheal resection for congenital tracheal stenosis. While this approach avoids the need for pulmonary artery suturing, it is not suitable for patients with tracheal stenosis repaired by pericardial patch tracheoplasty or those without significant tracheal narrowing. Criticism of this technique has been raised by Backer et al. (1), highlighted the potential risk of anterior compression of the trachea or left mainstem bronchus if the left pulmonary artery is positioned in front of the trachea. In our case, we confirmed that the tracheal obstruction pathology was eliminated after surgical intervention. Otherwise, we would have needed an additional tracheal surgery to our surgical method.

The complexity of coexisting anomalies, particularly those involving the heart and trachea, significantly impacts the prognosis for PAS patients. The studies have shown that postoperative mortality and morbidity are significantly increased in patients who underwent concurrent tracheal procedures (2, 6). However, the outcomes are generally favorable for children with PAS who do not require tracheal surgery. The important point emphasized in our case report is that in the face of this life-threatening clinical picture, with surgical intervention performed at the right time, immediate satisfactory results can be achieved with radical treatment.

## Conclusion

Pulmonary artery sling can present with progressively worsening respiratory distress, often leading to significant complications if not properly addressed. Effective treatment requires a comprehensive preoperative assessment followed by timely surgical intervention. Bronchoscopy plays a crucial role in evaluating tracheal compression and guiding the surgical approach. It is essential to highlight that both preoperative and postoperative care necessitate careful monitoring and coordination among a multidisciplinary team. Early surgical intervention in symptomatic patients remains the most effective approach for managing PAS, yielding favorable outcomes, and improving the patient's condition in the immediate postoperative period.

## Data Availability

The original contributions presented in the study are included in the article/Supplementary Material, further inquiries can be directed to the corresponding author/s.

## References

[1] Yong MS d'Udekem Y Brizard CP Robertson T Robertson CF Weintraub R Surgical management of pulmonary artery sling in children. J Thorac Cardiovasc Surg. (2013) 145(4):1033–9. 10.1016/j.jtcvs.2012.05.017 22698556

[2] Backer CL Idriss FS Holinger LD Mavroudis C. Pulmonary artery sling. Results of surgical repair in infancy. J Thorac Cardiovasc Surg. (1992) 103(4):683–91. 1548911

[3] Fiore AC Brown JW Weber TR Turrentine MW. Surgical treatment of pulmonary artery sling and tracheal stenosis. Ann Thorac Surg. (2005) 79(1):38–46; discussion 38–46. 10.1016/j.athoracsur.2004.06.005 15620911

[4] Susanti DS Wardoyo S Makdinata W Monika AS Billy M Ulfalian A. Left pulmonary artery sling repair without cardiopulmonary bypass: a case report. Int J Surg Case Rep. (2024) 118:109692. 10.1016/j.ijscr.2024.109692 38669803 PMC11066423

[5] Jonas RA Spevak PJ McGill T Castaneda AR. Pulmonary artery sling: primary repair by tracheal resection in infancy. J Thorac Cardiovasc Surg. (1989) 97(4):548–50. 2486055

[6] Oshima Y Yamaguchi M Yoshimura N Sato S Muraji T Nishijima E Management of pulmonary artery sling associated with tracheal stenosis. Ann Thorac Surg. (2008) 86(4):1334–8. 10.1016/j.athoracsur.2008.04.020 18805188

